# On Poisoning with Arsenic

**Published:** 1817-08

**Authors:** James Kerr


*For the London Medical and Physical Journal.*

### *On Poisoning with Arsenic;* by JAMES KERR, Esq.

READING in the newspapers, in April last, an account of the very important trial of a surgeon, at Falmouth, on a charge of murder, I anxiously waited in expectation of seeing, in your valuable Journal, some remarks on a case in which the medical public is so much interested. But, as nothing has appeared except a notice of it under the head of Medical Intelligence, containing nothing conclusive, and in which the experiments of Dr. Neale are not mentioned, I beg to submit to your readers a few remarks on the subject.

As the circumstances of the case may not be known to all your readers, I shall briefly state them. A surgeon, practising at Falmouth, was indicted for poisoning his mother-in-law, by giving her arsenic. The principal witness for the prosecution was Dr. Edwards, of the same town, who, amongst other particulars, stated, that, upon trying the contents of the stomach with the test of sulphate of copper with carbonate of potash, a green precipitate took place; and on trying another portion with carbonate of potash and nitrate of silver, the yellow precipitate so well known was the result; that he had not a sufficient quantity to attempt the re-production of metallic arsenic, but he was quite satisfied with these experiments as determining the existence of arsenic.

Fortunately for the prisoner, Dr. Neale, of Exeter, was examined as a witness, who stated that he did not consider the above experiments as at all to be relied on, and then related the result of two experiments which appeared completely to invalidate those of Dr. Edwards, and, no doubt, considerably influenced the jury in their decision.

Dr. Neale also stated that he thought nothing short of the re-production of metallic arsenic could be satisfactory. Dr. Neale's experiments were as follow:

1st. To a solution of sulphate of copper a small quantity of decoction of onions was added; a solution of phosphate of soda dropped into this mixture, produced a green precipitate very much resembling the Scheele's green.

2d. To a solution of phosphate of soda was added some decoction of onions: on applying the nitrate of silver, a yellow precipitate took place, resembling that when a solution of arsenic is used.

There is one circumstance connected with the case of the deceased which greatly adds to the merit and applicability of these experiments, and that is, the deceased had dined on rabbit and *onion-sauce* a few hours before her death; and, as many of the animal fluids contain phosphate of soda, we readily perceive the source of each substance from which the analogy was taken.

A few days after reading the account of this interesting trial, I went over the experiments of Dr. Neale, and repeated them before three medical friends of this town, the results of which, with some observations, I shall now state.

In Dr. Neale's first experiment, I found the green precipitate so exactly resembling that from a solution of arsenic, that I was induced afterwards to repeat it on a larger scale, so as to obtain a small lump of it in a dried state, and then the resemblance to the Scheele's green was remarkably striking. In his second experiment the result is so much like that made with arsenic, that it would puzzle most people to distinguish them; if there be any difference, it is that, when the phosphate of soda is employed, the precipitate is of a more beautiful sulphur colour than when arsenic is used. It may be proper to mention, that, in this experiment, the decoction of onions is not at all necessary, its addition or omission making no difference in the colour of the precipitate; but, in the first experiment it is indispensable, the peculiar green precipitate not taking place till it be added.

As a further elucidation of the subject, I took the lump of green precipitate before mentioned, and laid it on a red hot iron, holding over it a plate of polished copper: it soon became a black cinder, without at all affecting the copper. On substituting a lump of arsenite of copper, or Scheele's green, white fumes are exhaled, and the copper plate is immediately covered with a white coat, in the same manner as when white arsenic is employed.

The conclusions, to be drawn from these experiments appear to me to be of considerable importance to the public public in general. We now see two experiments, which, though not considered quite conclusive, were thought to be strongly corroborative, rendered completely nugatory. In your Journal for December, 1815, is a paper by Mr. Crowfoot, in which, after trying the two experiments made by Dr. Edwards, and that with the copper plates, the writer says, "From the whole, therefore, though every experiment produced conviction of the fact, it may be useful to state that the weakest proof was that by the action of heat, and the strongest and most decisive by the *admirable test of Mr. Hume.*" Yet who will place confidence in this test in future? I hope this will not be understood as any censure of Mr. Crowfoot. So far from any intention of that kind, I take this opportunity of stating that I read Mr. Crowfoot's communication at the time with great pleasure, and had not the least doubt of its accuracy. What surprises me is, that Mr. Hume and other excellent chemists should not have been acquainted with the fact that phosphate of soda produces with the nitrate of silver the same beautiful yellow precipitate that arsenite of potash does. In searching for information on this subject, I found, in your Journal for 1816, under the head of Medical and Physical Intelligence, that "an ingenious student at Guy's Hospital" had suggested this fact to Dr. Marcet; but, what is very curious, he goes on to say, "Yet in juridical cases other tests may be requisite to be assured of the presence of arsenic. The addition of sulphate of copper and potash, and the formation of Scheele's green, affords *a very satisfactory confirmation.*" After the first experiment of Dr. Neale, I am inclined to think this "ingenious student" will have his confidence shaken in this test as well as in the other. In consulting the celebrated work of M. Orfila on Toxicology, I cannot find that he was acquainted with either of these facts. In a note to page 113 of vol. i. part i. he says, "It may readily be conceived that the nitrate of silver lately proposed by Mr. Home (Hume) for discovering the arsenious acid, ought to be a very uncertain test in a great number of cases. In fact, if the quantity of arsenious acid mixed with the aliments is very small, and these contain muriates, there ought to be formed, at the same time, a small quantity of arsenite of silver of a yellow colour, and a great deal of muriate of silver of a white; in such manner that the precipitate would appear of this last colour, whilst it ought properly to be yellow." Here it is evident that, had M. Orfila known that phosphate of soda would produce the same yellow precipitate, he would have mentioned it. Nor can I find any mention of a green precipitate produced in any way, but by the help of arsenious acid, or white arsenic.

I have

I have thus endeavoured to shew how much obligation we are under to Dr. Neale; for, although previous mention was made of the yellow precipitate, I believe he was the first to inform us that an apple-green precipitate resembling the arsenite of copper or Scheele's green could be produced without the existence of arsenic.

*Great Yarmouth;*
*June* 12, 1817.

---

*For the London Medical and Physical Journal.*

